# Fuzzy Risk Evaluation in Failure Mode and Effects Analysis Using a D Numbers Based Multi-Sensor Information Fusion Method

**DOI:** 10.3390/s17092086

**Published:** 2017-09-12

**Authors:** Xinyang Deng, Wen Jiang

**Affiliations:** School of Electronics and Information, Northwestern Polytechnical University, Xi’an 710072, China

**Keywords:** fuzzy risk evaluation, failure mode and effects analysis, multi-sensor information fusion, D numbers, dempster-shafer evidence theory, fuzzy uncertainty

## Abstract

Failure mode and effect analysis (FMEA) is a useful tool to define, identify, and eliminate potential failures or errors so as to improve the reliability of systems, designs, and products. Risk evaluation is an important issue in FMEA to determine the risk priorities of failure modes. There are some shortcomings in the traditional risk priority number (RPN) approach for risk evaluation in FMEA, and fuzzy risk evaluation has become an important research direction that attracts increasing attention. In this paper, the fuzzy risk evaluation in FMEA is studied from a perspective of multi-sensor information fusion. By considering the non-exclusiveness between the evaluations of fuzzy linguistic variables to failure modes, a novel model called D numbers is used to model the non-exclusive fuzzy evaluations. A D numbers based multi-sensor information fusion method is proposed to establish a new model for fuzzy risk evaluation in FMEA. An illustrative example is provided and examined using the proposed model and other existing method to show the effectiveness of the proposed model.

## 1. Introduction

Failure mode and effects analysis (FMEA) is a widely used technology in many fields to identify potential failures or errors and further improve the reliability of systems by avoiding the occurrence of these failures or errors [1,2,3]. Risk evaluation is a crucial step in FMEA, which aims to identify failure modes with high risk so as to perfect system design to eliminating the risk [4,5]. In FMEA, risk priority number (RPN) approach is a classical method for the risk evaluation [6,7]. Since having clear physical meaning and easy to implement, the RPN approach has been received extensively concern and application. However, there still are some shortcomings in the RPN approach [8,9], for example the possible missing of risk factors, without considering the relative importance of risk factors, and so on. Among these drawbacks, failing to address the uncertainty in risk evaluation is one of the most concerned, and has attracted increasing attention [10,11,12,13].

In the risk evaluation of FMEA, domain experts’ knowledge and evaluations play a very importance role. Because there are human being’s judgments, it inevitably involves various types of uncertainties such as ignorance and fuzziness. Fuzzy set theory [14] provides a useful framework to describe the uncertain information. Therefore, risk evaluation under fuzzy environment, also known as fuzzy risk evaluation, has become an important research direction in FMEA. Many technologies have been developed to solve the problem of fuzzy risk evaluation in FMEA. Chin et al. [15] presented a data envelopment analysis (DEA) based FMEA to determine the risk priorities of failure modes. Jee et al. [16] proposed a fuzzy inference system (FIS)-based RPN model for the prioritization of failures. In [17], the authors have given applied a model of evolving tree to allow the failure modes in FMEA to be clustered and visualized. Reference [18] gives a detained literature review to the risk evaluation approaches in FMEA. By summarizing the existing approaches [10,18], one of the main branches is to regard the fuzzy risk evaluation in FMEA as a multiple criteria decision making (MCDM) problem under fuzzy environment. Many MCDM or multi-sensor information fusion technologies [19,20,21,22,23,24,25,26] have been used in FMEA, such as TOPSIS [27], VIKOR [28], evidential reasoning [29], and so on.

In this paper, we address the fuzzy risk evaluation in FMEA from a perspective of multi-sensor information fusion. Each risk factor in FMEA is regarded as a sensor or information source that yields an evaluation regarding the risk of each failure mode. Then, the risk evaluation of every failure mode therefore becomes the process of fusing these evaluations generated from the information sources that correspond to risk factors. Different from existing multi-sensor information fusion method used in FMEA, in this paper the non-exclusiveness between the evaluations of fuzzy linguistic variables to failure modes is taken into consideration. A novel model called D numbers [30,31,32,33] which is an extension of Dempster-Shafer evidence theory [34,35] is used to model the non-exclusive fuzzy evaluations. At first, a new D numbers based multi-sensor information fusion method is proposed. Then, the proposed multi-sensor information fusion method is applied to FMEA, which results in a novel model for fuzzy risk evaluation. At last, an illustrative example is given to demonstrate the effectiveness of the proposed model.

The rest of the paper is organized as follows. Section 2 gives a brief introduction about fuzzy set theory, RPN approach, as well as Dempster-Shafer evidence theory and D numbers. In Section 3, a novel multi-sensor information fusion method is proposed based on D numbers. Then, the new model for fuzzy risk evaluation in FMEA is presented in Section 4. Section 5 gives an illustrative example of the proposed model to show its effectiveness. Lastly, Section 6 concludes this paper. In addition, the notations of this paper are briefly introduced here: $\tilde{A}$ represents a fuzzy set or fuzzy number, and $\mu_{\tilde{A}}$ is its corresponding membership function, and ${Area}_{\tilde{A}}$ represents the area of $\tilde{A}$ in the graph; Ω and Θ stand for the frame of discernment in Dempster-Shafer theory and D numbers respectively, *m* represents a mass function and *D* is a D number; $u_{\neg E}\left(\tilde{A},\tilde{B}\right)$ is the non-exclusive degree between $\tilde{A}$ and $\tilde{B}$; BetP represents a distribution of pignistic probabilities; $C_{\tilde{F}}$ is the defuzzified value of fuzzy number $\tilde{F}$.

## 2. Preliminaries

### 2.1. Fuzzy Set Theory

Fuzzy set theory was first introduced by Zadeh [14] in 1965 to deal with the uncertainty information. In some real application environments, the states are subjective concepts which are too complex or too ill-defined to be reasonably described in conventional quantitative expressions. In those situation, fuzzy set theory provides an efficiently simple way to express the vagueness or imprecise information [36,37].

**Definition** **1.** *Let X be the universe of discourse, a fuzzy set $\tilde{A}$ is characterized by a membership function $\mu_{\tilde{A}}$ satisfying*

(1) $$\mu_{\tilde{A}}:X\to \left[0,1\right]$$

*where $\mu_{\tilde{A}}\left(x\right)$ is called the membership degree of x∈X belonging to fuzzy set $\tilde{A}$.*

**Figure 1:**
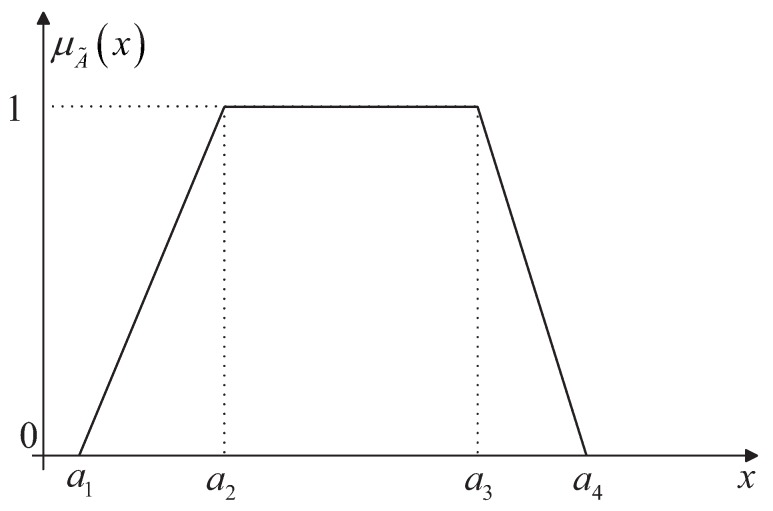
Graphically presentation of the trapezoidal fuzzy number.

For a finite set $A=\left\{x_{1},\ldots ,x_{i},\ldots ,x_{n}\right\},$, the fuzzy set $\left(\tilde{A},\mu_{\tilde{A}}\right)$ is often denoted by {μA˜(x1)x1,…,μA˜(xi)xi,…,μA˜(xn)xn}. It is easily found that a fuzzy set is described entirely by its membership function. When $\mu_{\tilde{A}}$ takes value from {0,1}, fuzzy set $\tilde{A}$ degenerates into a classical set. A fuzzy number $\tilde{A}$ is a fuzzy subset of the real number R, and its membership function is

(2) $$\mu_{\tilde{A}}\left(x\right):R\to \left[0,1\right]$$

where *x* is a real number and there definitely exists an element $x_{0}$ such that $\mu_{\tilde{A}}\left(x_{0}\right)=1$. Triangular and trapezoidal fuzzy numbers are the most widely used fuzzy numbers, and the former can be regarded as the special case of the latter. A trapezoidal fuzzy number is usually denoted as $\tilde{A}=\left(a_{1},a_{2},a_{3},a_{4}\right)$, as graphically shown in Figure 1, which has the following membership function

(3) $$\mu_{\tilde{A}}\left(x\right)=\left\{\begin{array}{cc} 0, & x\leq a_{1};\\ \frac{x-a_{1}}{a_{2}-a_{1}} & a_{1},\leq x\leq a_{2};\\ 1, & a_{2}\leq x\leq a_{3};\\ \frac{a_{4}-x}{a_{4}-a_{3}}, & a_{3}\leq x\leq a_{4};\\ 0, & a_{4}\leq x. \end{array}\right.$$

In theory and practice, fuzzy numbers are usually associated with linguistic variables to express the fuzzy evaluation to objects. A linguistic variable is a variable whose values are represented by words or sentences in a natural or artificial language, for example “Very Low”, “Low”, “Medium”, “High”, “Very High”, where there values are usually expressed by fuzzy numbers.

### 2.2. Risk Priority Number Approach in FEMA

Risk priority number (RPN) approach is a traditional risk evaluation method in FEMA. In this approach, the risk priority of each failure mode is expressed by a RPN which is defined by the following formula

(4) RPN=O×S×D

where *O* is the probability of occurrence of a failure mode, *S* is the severity of the failure effect, and *D* is the probability of a failure mode not being detected before it occurs. In the RPN approach, each factor among *O*, *S*, and *D* is evaluated by 10 rankings, as shown in Table 1, Table 2 and Table 3. The larger the RPN value, the higher the risk priority of a failure mode.

Although the RPN approach is easy to use, but it still has some shortcomings that are criticized in many studies [8,9]. For example, three risk factors O, S, D are considered with equal importance; Traditional RPN approach only considers three factors but ignores other possible influential factors to different application environment; Various sets of O, S and D may produce an identical RPN value, but their hidden risk implications may be different. Please refer to [8,9] for more details on the drawbacks of RPN approach.

### 2.3. Dempster-Shafer Evidence Theory and D Numbers

Dempster-Shafer evidence theory [34,35], also called Dempster-Shafer (D-S) theory or evidence theory, is a popular theory to deal with uncertain information [40,41,42,43,44,45]. Compared with traditional probability theory, this theory has an advantage of directly expressing the “uncertainty” by assigning the probability to the set composed of multiple objects, it therefore has attracted increasing interest in uncertainty reasoning and modelling [46,47,48,49,50,51,52] and been further extended such as generalized evidence theory (GET) in open world [53,54,55].

Let Ω be a set of mutually exclusive and collectively exhaustive events, indicated by $\Omega =\{E_{1},E_{2},\ldots ,E_{i},\ldots ,E_{N}\}$, where set Ω is called a frame of discernment (FOD). The power set of Ω is indicated by $2^{\Omega }$, namely $2^{\Omega }=\left\{\emptyset ,\left\{E_{1}\right\},\ldots ,\left\{E_{N}\right\},\left\{E_{1},E_{2}\right\},\ldots ,\left\{E_{1},E_{2},\ldots ,E_{i}\right\},\ldots ,\Omega \right\}$. In D-S theory, the uncertain information is modelled by mass functions.

**Definition** **2.** *Given a FOD* Ω, *a mass function is a mapping $m:\;2^{\Omega }\to \left[0,1\right]$, such that*

(5) $$\begin{array}{c} m\left(\emptyset \right)=0\;and\;\sum_{A\in 2^{\Omega }}m(A)=1 \end{array}$$

*where set A with m(A)>0 is called a focal element, and the assigned m(A) measures the belief exactly assigned to A and represents how strongly the evidence supports A. A mass function is also called a basic probability assignment (BPA).*

Considering two pieces of evidence indicated by $m_{1}$ and $m_{2}$, Dempster’s rule of combination can be used to combine them. This rule assumes that these mass functions are mutually independent. The Dempster’s rule of combination, denoted by $m=m_{1}⊕m_{2}$, is defined as follows:

(6) $$m\left(A\right)=\left\{\begin{array}{cc} \frac{1}{1-K}\sum_{B\cap C=A}m_{1}\left(B\right)m_{2}\left(C\right)\;, & A\neq \emptyset \\ 0\;, & A=\emptyset \end{array}\right.$$

with

(7) $$K=\sum_{B\cap C=\emptyset }m_{1}\left(B\right)m_{2}\left(C\right).$$

In order to make decision in terms of a mass function [56], an approach, called pignistic probability transformation (PPT), is proposed by Smets and Kennes [57] to derive a probability distribution from a mass function. Let *m* be a mass function or BPA on FOD Ω, a PPT function $BetP_{m}:\Omega \to \left[0,1\right]$ associated to *m* is defined by

(8) $${BetP}_{m}\left(x\right)=\sum_{x\in A,A\subseteq \Omega }\frac{1}{\left|A\right|}\frac{m(A)}{1-m(\emptyset )}\;,$$

where m(∅)≠1 and |A| is the cardinality of proposition *A*.

Although D-S theory provides a good framework for uncertainty reasoning, this theory is also constrained by many strong hypotheses and hard constraints which limit its development and application to a large extend. For one hand, the elements in the FOD are required to be mutually exclusive. It is called exclusiveness hypothesis. For another, the sum of basic probabilities of a mass function must be equal to 1, which is called completeness constraint. To overcome these existing shortcomings in D-S theory and enhance its capability in expressing uncertain information, a novel model, named as D numbers, has been proposed recently [30,31,32,33,58]. D numbers relax mass function’s exclusiveness hypothesis and BPA’s completeness constraint.

**Definition** **3.** *Let $\Theta =\{F_{1},F_{2},\ldots ,F_{N}\}$ be a nonempty set satisfying $F_{i}\neq F_{j}$ if i≠j, ∀i,j={1,…,N}, a D number is a mapping formulated by*

(9) $$D:2^{\Theta }\to \left[0,1\right]$$

*with*

(10) $$\begin{array}{c} \sum_{B\subseteq \Theta }D(B)\leq 1\;and\;D\left(\emptyset \right)=0 \end{array}$$

*where* ∅ *is the empty set and B is a subset of* Θ.

If $\sum_{B\subseteq \Theta }D(B)=1$, the information is said to be complete; if $\sum_{B\subseteq \Theta }D(B)<1$, the information is said to be incomplete. If a D number is of complete information, it means that the D number is generated from an environment with the close-world assumption. By contrast, a D number with incomplete information is corresponding to the open-world assumption. With respect to the open-orld assumption, Smets [57] proposed a transferable belief model (TBM) which allows m(∅)>0. Compared with the TBM, in D numbers the open-world environment is implemented by letting $\sum_{B\subseteq \Theta }D(B)<1$. What’s more important, in the TBM each mass function is defined on a FOD which requires the internal elements are mutually exclusive. However, in D numbers the exclusiveness hypothesis is removed, i.e., the elements in Θ do not require mutual exclusiveness for D numbers.

## 3. Proposed Multi-Sensor Information Fusion Method Based on D Numbers

Let us consider a multiple criteria decision making (MCDM) problem, where each criterion can be regarded as an independent sensor or information source. Therefore, the process of resolving the MCDM problem can be treated as a process of multi-sensor information fusion. Assume there are *p* alternatives, indicated by $A_{i}$, i=1,…,p, and *q* criteria, denoted as $C_{j}$, j=1,…,q, and the weight of each criterion is $w_{j}$, j=1,…,q. Due to the uncertainty of decision-making environment, the evaluation to alternative $A_{i}$ on criterion $C_{j}$ is expressed as a D number indicated by $D_{ij}$, thus the decision matrix is represented as

(11) $$M=\begin{array}{cc} & \begin{array}{ccc} C_{1} & \ldots & C_{q} \end{array}\\ \begin{array}{c} A_{1}\\ \vdots \\ A_{p} \end{array} & \left(\begin{array}{ccc} D_{11} & \ldots & D_{1q}\\ \ldots & \ldots & \ldots \\ D_{p1} & \ldots & D_{pq} \end{array}\right) \end{array}.$$

In this paper, we assume that each evaluation $D_{ij}$ in the decision matrix *M* is information-complete, i.e., $\sum_{k}D_{ij}\left(B_{k}\right)=1$ for any i=1,…,p and j=1,…,q. Now the overall objective is to find the best alternative according to the decision matrix *M* and criteria’s weights mentioned above. In this study, we develop a new multi-sensor information fusion method based on D numbers to solve that problem. The key points of the proposed approach are presented as follows.

### 3.1. Non-Exclusiveness in D Numbers

Since the evaluations are in the form of D numbers and the theory of D numbers is found on the basis of non-exclusiveness assumption, the first step is to calculate the non-exclusive degrees in D numbers. The non-exclusiveness is the opposite of exclusiveness, representing a potential connection between elements in D numbers framework. By contrast, the exclusiveness refers to the characteristic that one object excludes the others, which is an either-or related thing but not the similarity.

**Definition** **4.** *Let $B_{i}$ and $B_{j}$ be two non-empty elements belonging to $2^{\Theta }$, the non-exclusive degree between $B_{i}$ and $B_{j}$ is characterized by a fuzzy membership function $u_{\neg E}$:*

(12) $$u_{\neg E}:2^{\Theta }\times 2^{\Theta }\to \left[0,1\right]$$

*with*

(13) $$u_{\neg E}\left(B_{i},B_{j}\right)=\left\{\begin{array}{c} 1,\;B_{i}\cap B_{j}\neq \emptyset \\ p,\;p\in \left[0,1\right],B_{i}\cap B_{j}=\emptyset \end{array}\right.$$

*and*

(14) $$u_{\neg E}\left(B_{i},B_{j}\right)=u_{\neg E}\left(B_{j},B_{i}\right).$$

If letting the exclusive degree between $B_{i}$ and $B_{j}$ be denoted as $u_{E}$, then $u_{E}=1-u_{\neg E}$.

In our previous study [59], a simple approach was proposed to determine the non-exclusive degrees in D numbers. In that approach it assumes that all non-exclusive degrees among elements in FOD Θ have already been determined, then each exclusive degree in the power set space $2^{\Theta }$ can be calculated by the following formula:

(15) $$u_{\neg E}\left(B_{i},B_{j}\right)=\max_{x\in B_{i},y\in B_{j}}\left\{u_{\neg E}\left(x,y\right)\right\},\;B_{i},B_{j}\in 2^{\Theta }.$$

An illustrative example regarding the calculation of non-exclusive degrees is given as follows.

**Figure 2:**
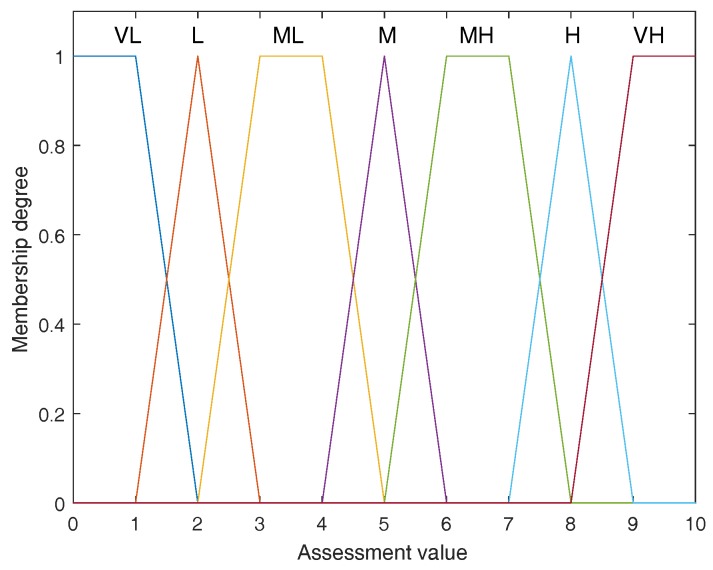
Graphically presentation of fuzzy linguistic variables in Table 4.

**Example** **1.** Supposing each evaluation in decision matrix M shown in Equation (11) is defined on a set of linguistic variables Θ={VL,L,ML,M,MH,H,VH} in which every linguistic variable is represented by a trapezoidal fuzzy number given in Table 4 and graphically presented as Figure 2.

*The set* Θ *is seen as the FOD. At first, let us calculate the non-exclusive degrees between elements in FOD* Θ. *In this paper, an approach based on fuzzy numbers’ areas is utilized. Assume the areas of fuzzy numbers $\tilde{A}$ and $\tilde{B}$ are respectively denoted as ${Area}_{\tilde{A}}$ and ${Area}_{\tilde{B}}$, and the area of the overlap of $\tilde{A}$ and $\tilde{B}$ is ${Area}_{\tilde{A}\cap \tilde{B}}$, then the non-exclusive degree between $\tilde{A}$ and $\tilde{B}$ is defined as*

(16) $$u_{\neg E}\left(\tilde{A},\tilde{B}\right)=\frac{{Area}_{\tilde{A}\cap \tilde{B}}}{{Area}_{\tilde{A}}+{Area}_{\tilde{B}}-{Area}_{\tilde{A}\cap \tilde{B}}}.$$

*According to Equation (16), each non-exclusive degree between elements in FOD* Θ *therefore can be obtained as shown in the following matrix*

$$\begin{array}{cc} & \begin{array}{ccccccc} \;VL & \;L & \;ML & \;M & \;MH & \;H & \;VH \end{array}\\ \begin{array}{c} VL\\ L\\ ML\\ M\\ MH\\ H\\ VH \end{array} & \left(\begin{array}{ccccccc} 1 & 0.1111 & 0 & 0 & 0 & 0 & 0\\ 0.1111 & 1 & 0.0909 & 0 & 0 & 0 & 0\\ 0 & 0.0909 & 1 & 0.0909 & 0 & 0 & 0\\ 0 & 0 & 0.0909 & 1 & 0.0909 & 0 & 0\\ 0 & 0 & 0 & 0.0909 & 1 & 0.0909 & 0\\ 0 & 0 & 0 & 0 & 0.0909 & 1 & 0.1111\\ 0 & 0 & 0 & 0 & 0 & 0.1111 & 1 \end{array}\right) \end{array}$$

*Having the above non-exclusive degree matrix of between elements in* Θ, *according to Equation (15), we can easily calculate the non-exclusive degree of any pair of elements in $2^{\Theta }$. For example, as for {L} and {ML,M}, we have*

$$\begin{array}{c} u_{\neg E}\left(\left\{L\right\},\left\{ML,M\right\}\right)=\max\left\{u_{\neg E}\left(\left\{L\right\},\left\{ML\right\}\right),u_{\neg E}\left(\left\{L\right\},\left\{M\right\}\right)\right\}\\ \;\;\;\;\;\;\;\;\;=\max\left\{0.0909,0\right\}\\ \;\;\;\;\;\;\;\;\;=0.0909. \end{array}$$

### 3.2. Fusing the Evaluations to the Same Alternative on Different Criteria

In order to implement the overall assessment to each alternative, all evaluations belonging to the same alternative on different criteria should be combined according to the perspective of multi-sensor information fusion. In this paper since the evaluations are given in the form of D numbers, it becomes the fusion of D numbers. In our recent study [59], a D numbers combination rule (DNCR) has been proposed from a perspective of conflict redistribution. The proposed DNCR is shown as follows.

**Definition** **5.** *Let $D_{1}$ and $D_{2}$ be two D numbers defined on* Θ *with $\sum_{B\subseteq \Theta }D_{1}\left(B\right)=1$ and $\sum_{C\subseteq \Theta }D_{2}\left(C\right)=1$, the combination of $D_{1}$ and $D_{2}$, indicated by $D=D_{1}⊙D_{2}$, is defined by*

(17) D(A)=0,A=∅11−KD∑B∩C=Au¬E(B,C)D1(B)D2(C)+∑B∪C=AB∩C=∅u¬E(B,C)D1(B)D2(C),A≠∅

*with*

(18) $$K_{D}=\sum_{B\cap C=\emptyset }\left(1-u_{\neg E}\left(B,C\right)\right)D_{1}\left(B\right)D_{2}\left(C\right).$$

The above rule for D numbers is a generalization of Dempster’s rule for the model of D numbers, because it can totally reduce to the classical Dempster’s rule when $u_{\neg E}\left(B,C\right)=0$ for any B∩C=∅. Different from the D-S theory, in this rule the impact of of non-exclusiveness in D numbers is taken into consideration.

Although the rule defined in Definition 5 provides a solution for the combination of D numbers, it must point out that such rule does not preserve the associative property, i.e., $\left(D_{1}⊙D_{2}\right)⊙D_{3}\neq D_{1}⊙\left(D_{2}⊙D_{3}\right)\neq \left(D_{1}⊙D_{3}\right)⊙D_{2}$, and it is only suitable for the combination of two D numbers. In order to implement the effective combination of multiple D numbers, a novel combination rule for multiple D numbers is developed in this paper by utilizing the idea of induced ordered weighted averaging (IOWA) operator [60] which imports an order variable compared with other aggregation operators [61,62].

**Definition** **6.** *Let $D_{1}$, $D_{2}$, ⋯, $D_{n}$ be n D numbers, and $v_{j}$ be an order variable for each $D_{j}$, j=1,…,n, therefore each piece of information is indicated by tuple $<v_{j},D_{v_{j}}>$. The combination operation of these information represented by D numbers is defined as a mapping ${Agg}_{D}$, such that*

(19) $${Agg}_{D}\left(D_{1},D_{2},\ldots ,D_{n}\right)=\left[\ldots \left[D_{\lambda_{1}}⊙D_{\lambda_{2}}\right]⊙\ldots ⊙D_{\lambda_{n}}\right]$$

*where $D_{\lambda_{i}}$ is the corresponding $D_{v_{j}}$ in tuple $<v_{j},D_{v_{j}}>$ having the i-th largest order variable $v_{j}$.*

In this paper, for the MCDM problem the weight of each criterion is regarded as the order variable of corresponding D numbers so as to fuse the evaluations to each alternative on multiple criteria. For each alternative $A_{i}$, i=1,…,p, the obtained aggregated evaluation is denoted as $D_{i}$ which is defined over the FOD Θ consisting of fuzzy linguistic variables.

### 3.3. Decision-Making Based on the Aggregated Evaluations under Fuzzy Environment

In this paper, each aggregated evaluation is also a D number, indicated by $D_{i}$, i=1,…,p, which is defined on FOD Θ composed by fuzzy linguistic variables. Assume $\Theta =\{\theta_{t},t=1,\ldots ,l\}$, and each element $\theta_{t}$ is represented by a trapezoidal fuzzy number $\theta_{t}=\left(a_{t1},a_{t2},a_{t3},a_{t4}\right)$. Each $D_{i}$ is firstly transformed to a distribution of pignistic probabilities, denoted as ${BetP}_{i}$, by means of the PPT as follows

(20) $${BetP}_{i}\left(\theta_{t}\right)=\sum_{\theta_{t}\in B,B\subseteq \Theta }\frac{D_{i}\left(B\right)}{\left|B\right|},\;t=1,\ldots ,l.$$

Once the ${BetP}_{i}$ is obtained, it then be transformed to a fuzzy aggregated evaluation $\tilde{F_{i}}$ to express the overall assessment to alternative *i*, represented as

(21) $$\tilde{F_{i}}=\left(f_{i1},f_{i2},f_{i3},f_{i4}\right),\;i=1,\ldots ,q$$

in which

(22) $$f_{ik}=\sum_{t=1}^{l}BetP_{i}\left(\theta_{t}\right)\times a_{tk},\;k=1,2,3,4.$$

At last, these fuzzy aggregated evaluation $\tilde{F_{i}}$, i=1,…,p, are converted to crisp values through a defuzzification process in order to rank all alternatives. Among existing defuzzification techniques, the centroid defuzzification approach is a common used one. Given a fuzzy number $\tilde{F}$ with membership function $\mu_{\tilde{F}}\left(x\right)$, in terms of the centroid defuzzification approach we can have

(23) $$C_{\tilde{F}}=\frac{\int x\mu_{\tilde{F}}\left(x\right)dx}{\int \mu_{\tilde{F}}\left(x\right)dx},$$

where $C_{\tilde{F}}$ is the defuzzified value of $\tilde{F}$. In terms of the study in [63], while $\tilde{F}$ is a trapezoidal fuzzy number indicated by $\left(f_{1},f_{2},f_{3},f_{4}\right)$ the centroid-based defuzzified value turns out to be

(24) $$C_{\tilde{F}}=\frac{1}{3}\left(f_{1}+f_{2}+f_{3}+f_{4}-\frac{f_{3}f_{4}-f_{1}f_{2}}{\left(f_{3}+f_{4}\right)-\left(f_{1}+f_{2}\right)}\right).$$

Via the defuzzification process, for each fuzzy aggregated evaluation $\tilde{F_{i}}$, a defuzzified value $C_{{\tilde{F}}_{i}}$ can be derived. The best alternative is finally determined by finding the one with the largest defuzzified value.

## 4. The Proposed Model for Fuzzy Risk Evaluation in FMEA

**Figure 3:**
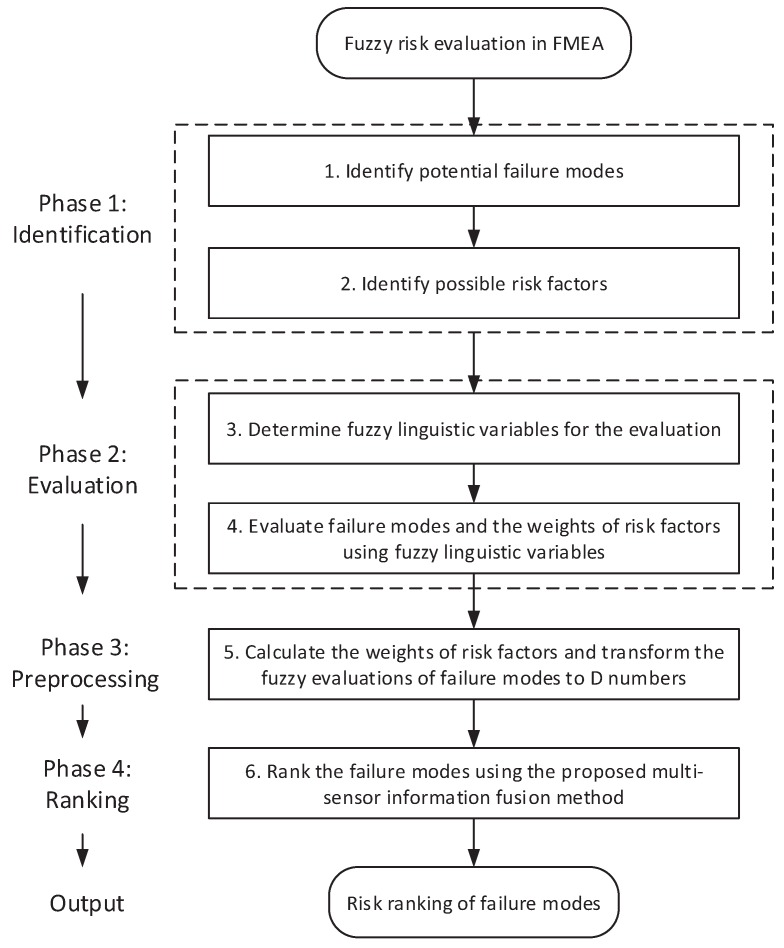
Flowchart of the proposed model for fuzzy risk evaluation in FMEA.

In terms of the multi-sensor information fusion method based on D numbers as presented above, a novel model for fuzzy risk evaluation in FMEA is proposed in this section. The flowchart of the proposed model is shown in Figure 3, which briefly contains four phases, namely identification, evaluation, preprocessing and ranking. The final output is the risk ranking of potential failure modes.

- Step 1: Identify all potential failure modes according to the practical FMEA environment.
- Step 2: Identify all possible risk factors for the risk evaluation task. In the RPN approach, the risk factors are probability of occurrence (O), severity of failure effect (S), and probability of a failure mode not being detected (D).
- Step 3: Determine fuzzy linguistic variables for the evaluation including evaluating failure modes and evaluating the weights of risk factors.
- Step 4: Evaluate failure modes and the weights of risk factors using fuzzy linguistic variables determined in the above step. These are usually given by domain experts of FMEA risk evaluation.
- Step 5: Calculate the weight of each risk factor and transform the fuzzy evaluations of failure modes on different risk factors to D numbers. This is a process of data preprocessing.
- Step 6: Rank the failure modes using the proposed multi-sensor information fusion method in above section so as to generate the risk ranking of all failure modes.

## 5. Illustrative Example

In the section, an illustrative example is given to show the effectiveness of the proposed model for fuzzy risk evaluation in FMEA. This example is original from literature [28]. In [28], the authors developed an extended VIKOR method for risk evaluation in FMEA under fuzzy environment. In this paper, we will solve the problem by using our proposed model and compare the obtained result with that of literature [28].

Step 1: Identify all potential failure modes. By following literature [28], a hospital wants to rank the most serious failure modes during general anesthesia process, and six potential failure modes are identified which are denoted as FM 1, FM 2, FM 3, FM 4, FM 5, FM 6.

Step 2: Identify all possible risk factors. In this application, the risk factors are consistent with the RPN approach, therefore there are three risk factors, namely O, S, D.

Step 3: Determine fuzzy linguistic variables for the evaluation. As for the evaluation of failure modes, a set of linguistic variables including Very Low (VL), Low (L), Medium Low (ML), Medium (M), Medium High (MH), Very High (VH) is used as shown in Table 4. In addition, for the evaluation of risk factors’ weights, the fuzzy linguistic variables are given in Table 5.

Step 4: Evaluate failure modes and the weights of risk factors using fuzzy linguistic variables. As given in literature [28], a FMEA team of five decision makers, DM 1, DM 2, DM 3, DM 4, DM 5, is employed to evaluate failure modes and the weights of risk factors. With respect to risk factors’ weights, all five decision makers’ evaluations are given in Table 6. For the six failure modes, the evaluations from the FMEA team are given in Table 7.

Step 5: At this step, the weight of each risk factor is calculated at first. Since the calculation of risk factors’ weights is not the core concern of this paper, we simply continue to use the weights obtained in literature [28]. The importance of O is 0.768, and S 0.878, and D 0.650, therefore the weights of these risk factors are $w_{O}=0.768/\left(0.768+0.878+0.650\right)=0.3345$, $w_{S}=0.878/\left(0.768+0.878+0.650\right)=0.3824$, $w_{D}=0.650/\left(0.768+0.878+0.650\right)=0.2831$. Secondly, let us transform the fuzzy evaluations of failure modes on risk factors to D numbers. In this example since multiple decision makers are included so as to form a group decision making environment, we use the proportion of each evaluation to construct the D numbers. For example, for FM 1 on risk factor O, five decision makers respectively give evaluations M, M, M, MH, M, hence we can construct a D number D(M)=0.8, D(MH)=0.2. In terms of this means, the evaluations to failure modes are transformed to the form of D numbers, as shown in Table 8.

Step 6: Rank the failure modes using the proposed multi-sensor information fusion method. At first, the fuzzy linguistic variables in Table 4 form a FOD Θ={VL,L,ML,M,MH,H,VH}. Each exclusive degree between elements in Θ has been obtained in Example 1. According to Equation (15), the non-exclusive degree of any pair of elements in $2^{\Theta }$ can be easily obtained. Secondly, for every failure mode the evaluations on O, S, and D can be fused based on the proposed combination operation in Definition 6. For FM 1, the aggregated evaluation is

$$\begin{array}{c} D_{1}\left(\left\{M\right\}\right)=0.558,\\ D_{1}\left(\left\{ML\right\}\right)=0.263,\\ D_{1}\left(\left\{ML,M\right\}\right)=0.175,\\ D_{1}\left(\left\{ML,M,MH\right\}\right)=0.004. \end{array}$$

For FM 2,

$$\begin{array}{c} D_{2}\left(\left\{M,MH\right\}\right)=0.702,\\ D_{2}\left(\left\{M,MH,H\right\}\right)=0.298. \end{array}$$

For FM 3,

$$\begin{array}{c} D_{3}\left(\left\{MH\right\}\right)=0.943,\\ D_{3}\left(\left\{M,MH\right\}\right)=0.057. \end{array}$$

For FM 4,

$$\begin{array}{c} D_{4}\left(\left\{ML\right\}\right)=0.236,\\ D_{4}\left(\left\{ML,M\right\}\right)=0.756,\\ D_{4}\left(\left\{VL,L,ML\right\}\right)=0.008. \end{array}$$

For FM 5,

$$\begin{array}{c} D_{5}\left(\left\{ML\right\}\right)=0.178,\\ D_{5}\left(\left\{ML,M\right\}\right)=0.714,\\ D_{5}\left(\left\{ML,M,MH\right\}\right)=0.043,\\ D_{5}\left(\left\{L,ML,M\right\}\right)=0.065. \end{array}$$

For FM 6,

$$\begin{array}{c} D_{6}\left(\left\{M,MH,H\right\}\right)=1.000. \end{array}$$

Thirdly, by using the PPT, we have: ${BetP}_{1}\left(\left\{ML\right\}\right)=0.3516$, ${BetP}_{1}\left(\left\{M\right\}\right)=0.6471$, ${BetP}_{1}\left(\left\{MH\right\}\right)=0.0013$ for FM 1; ${BetP}_{2}\left(\left\{M\right\}\right)=0.4504$, ${BetP}_{2}\left(\left\{MH\right\}\right)=0.4504$, ${BetP}_{2}\left(\left\{H\right\}\right)=0.0993$ for FM 2; ${BetP}_{3}\left(\left\{M\right\}\right)=0.0286$, ${BetP}_{3}\left(\left\{MH\right\}\right)=0.9714$ for FM 3; ${BetP}_{4}\left(\left\{VL\right\}\right)=0.0026$, ${BetP}_{4}\left(\left\{L\right\}\right)=0.0026$, ${BetP}_{4}\left(\left\{ML\right\}\right)=0.6168$, ${BetP}_{4}\left(\left\{M\right\}\right)=0.3780$ for FM 4; ${BetP}_{5}\left(\left\{L\right\}\right)=0.0216$, ${BetP}_{5}\left(\left\{ML\right\}\right)=0.5712$, ${BetP}_{5}\left(\left\{M\right\}\right)=0.3928$, ${BetP}_{5}\left(\left\{MH\right\}\right)=0.0144$ for FM 5; and ${BetP}_{6}\left(\left\{M\right\}\right)=0.3333$, ${BetP}_{6}\left(\left\{MH\right\}\right)=0.3333$, ${BetP}_{6}\left(\left\{H\right\}\right)=0.3333$ for FM 6. Fourthly, these pignistic probabilities are then transformed to fuzzy aggregated evaluations according to Equations (21) and (22) which are

$$\tilde{F_{1}}=\left(3.2982,4.2982,4.6511,5.6511\right),$$

$$\tilde{F_{2}}=\left(4.7482,5.7482,6.1986,7.1986\right),$$

$$\tilde{F_{3}}=\left(4.9714,5.9714,6.9429,7.9429\right),$$

$$\tilde{F_{4}}=\left(2.7480,3.7454,4.3648,5.3648\right),$$

$$\tilde{F_{5}}=\left(2.8072,3.8072,4.3928,5.3928\right),$$

$$\tilde{F_{6}}=\left(5.3333,6.3333,6.6667,7.6667\right).$$

**Figure 4:**
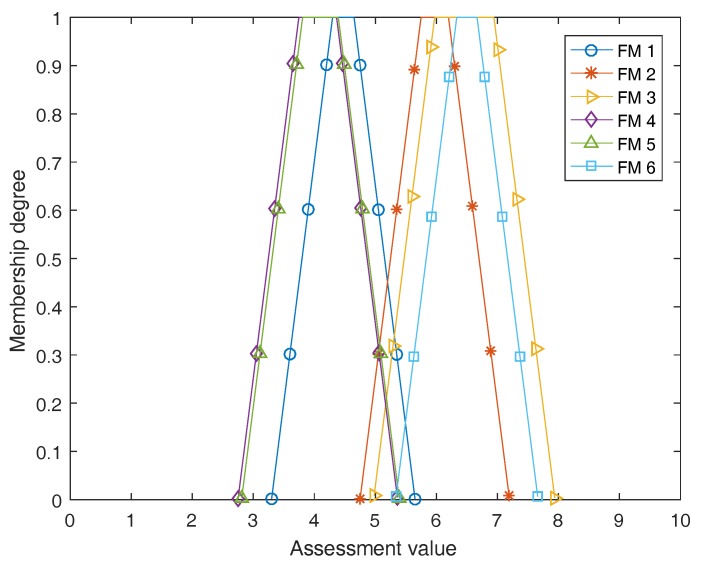
Graphically presentation of fuzzy aggregated evaluations.

These fuzzy aggregated evaluations are graphically shown in Figure 4. At last, in terms of the centroid defuzzification approach we can have $C_{{\tilde{F}}_{1}}=4.4746$, $C_{{\tilde{F}}_{2}}=5.9734$, $C_{{\tilde{F}}_{3}}=6.4571$, $C_{{\tilde{F}}_{4}}=4.0559$, $C_{{\tilde{F}}_{5}}=4.1000$, $C_{{\tilde{F}}_{6}}=6.5000$. Therefore, the risk ranking of all failure modes from high to low is FM6≻FM3≻FM2≻FM1≻FM5≻FM4. From the result, it is found that the failure mode with the highest risk is FM 6 and that having the lowest risk is FM 4.

The above steps have clearly shown the process of using the proposed model to do the risk evaluation in FMEA under fuzzy environment. Now we compare the result obtained by the proposed model with that from other method. In literature [28], Liu et al. dealt with the risk evaluation in FMEA with an extended VIKOR method. The results of the risk ranking are given in Table 9. In [28], the failure modes are ranked in terms of three indicators S, R, Q. By S, the failure modes with the highest and lowest risk are FM 3 and FM 4; By R, the failure modes having the highest and lowest risk are respectively FM 6 and FM 4; By Q, the failure modes with the highest and lowest risk are FM 3 and FM 4, respectively. Comparing the proposed model and the extended VIKOR method in [28], the ranking obtained by the proposed model is basically same with that of R. In addition, both the two methods have identified FM 4 is the failure mode of lowest risk. In addition, as a whole the failure modes can be classified into two groups by S, R, Q, and the first group which has higher risk is composed by FM 3, FM 6, FM 2, the second group having lower risk includes FM 1, FM 5, FM 4. By using the proposed model, we also obtain the same classification that FM 6, FM 3, FM 2 are in the group with higher risk and FM 1, FM 5, FM 4 constitute the group with lower risk. Through the above analysis and comparison, therefore it shows that the proposed model is effective for risk evaluation in FMEA.

## 6. Conclusions

In this paper, the risk evaluation of failure modes in FMEA has been studied in an environment involving fuzzy uncertainty. A novel model is proposed for the fuzzy risk evaluation in FMEA. Within the proposed model, a D numbers based multi-sensor information fusion method has been presented to evaluate potential failure modes and rank the risk levels of failure modes. Since the use of D numbers which is a new model of extending classical D-S theory, the non-exclusiveness between the evaluations of fuzzy linguistic variables has been taken into consideration in the proposed method. Compared with some existing risk evaluation methods in FMEA, the proposed method overcomes the shortcomings of traditional RPN approach to some degrees and obtains comparable performances relative to other MCDM technologies used in FMEA. This study provides a new solution for the risk evaluation in FMEA under fuzzy environment and it is especially suitable for the case that contains non-exclusive fuzzy evaluations. In the future research, on one hand we hope to seek more practical applications with read data to analyze the proposed D numbers based multi-sensor information fusion method, on the other hand we will explore other technologies such as Physarum-inspired model [64] to improve the risk evaluation in FMEA.

## Figures and Tables

**Table 1:** Assessment rankings for occurrence in FMEA [38,39].

Ranking	Probability of Occurrence	Possible Failure Rate
10	Extremely high: failure almost inevitable	≥1/2
9	Very high	1/3
8	Repeated failures	1/8
7	High	1/20
6	Moderately high	1/80
5	Moderate	1/400
4	Relatively low	1/2000
3	Low	1/15,000
2	Remote	1/150,000
1	Nearly impossible	≤1/1,500,000

**Table 2:** Assessment rankings for severity in FMEA [38,39].

Ranking	Effect	Severity of Effect
10	Hazardous without warning	Very high severity ranking when a potential failure mode affects safe vehicle operation and/or involves noncompliance with government regulations without warning
9	Hazardous with warning	Very high severity ranking when a potential failure mode affects safe vehicle operation and/or involves noncompliance with government regulations with warning
8	Very high	Vehicle/item inoperable, with loss of primary function
7	High	Vehicle/item operable, but at reduced level of performance. Customer dissatisfied
6	Moderate	Vehicle/item operable, but comfort/convenience item(s) inoperable. Customer experiences discomfort
5	Low	Vehicle/item operable, but comfort/convenience item(s) operable at reduced level of performance. Customer experiences some dissatisfaction.
4	Very low	Cosmetic defect in finish, fit and finish/squeak or rattle item that does not conform to specifications. Defect noticed by most customers
3	Minor	Cosmetic defect in finish, fit and finish/squeak or rattle item that does not conform to specifications. Defect noticed by average customer
2	Very minor	Cosmetic defect in finish, fit and finish/squeak or rattle item that does not conform to specifications. Defect noticed by discriminating customers
1	None	No effect

**Table 3:** Assessment rankings for detection in FMEA [38,39].

Ranking	Detection	Criteria
10	Absolutely impossible	Design control will not and/or cannot detect a potential cause/mechanism and subsequent failure mode; or there is no design control
9	Very remote	Very remote chance the design control will detect a potential cause/mechanism and subsequent failure mode
8	Remote	Remote chance the design control will detect a potential cause/mechanism and subsequent failure mode
7	Very low	Very low chance the design control will detect a potential cause/mechanism and subsequent failure mode
6	Low	Low chance the design control will detect a potential cause/mechanism and subsequent failure mode
5	Moderate	Moderate chance the design control will detect a potential cause/mechanism and subsequent failure mode
4	Moderately high	Moderately high chance the design control will detect a potential cause/mechanism and subsequent failure mode
3	High	High chance the design control will detect a potential cause/mechanism and subsequent failure mode
2	Very high	Very high chance the design control will detect a potential cause/mechanism and subsequent failure mode
1	Almost certain	Design control will almost certainly detect a potential cause/mechanism and subsequent failure mode

**Table 4:** Linguistic variables for evaluating the weights of risk factors.

Linguistic Variables	Fuzzy Numbers
Very Low (VL)	(0, 0, 1, 2)
Low (L)	(1, 2, 2, 3)
Medium Low (ML)	(2, 3, 4, 5)
Medium (M)	(4, 5, 5, 6)
Medium High (MH)	(5, 6, 7, 8)
High (H)	(7, 8, 8, 9)
Very High (VH)	(8, 9,10,10)

**Table 5:** Linguistic variables for the evaluation.

Linguistic Variables	Fuzzy Numbers
Very Low (VL)	(0, 0, 0.1, 0.2)
Low (L)	(0.1, 0.2, 0.2, 0.3)
Medium Low (ML)	(0.2, 0.3, 0.4, 0.5)
Medium (M)	(0.4, 0.5, 0.5, 0.6)
Medium High (MH)	(0.5, 0.6, 0.7, 0.8)
High (H)	(0.7, 0.8, 0.8, 0.9)
Very High (VH)	(0.8, 0.9, 1, 1)

**Table 6:** The evaluations to the weights of risk factors from the FMEA team.

Risk Factor	FMEA Team Member				
	DM 1	DM 2	DM 3	DM 4	DM 5
O	H	H	VH	H	MH
S	VH	VH	H	VH	VH
D	MH	MH	M	H	MH

**Table 7:** The evaluations to failure modes from the FMEA team.

	FM 1	FM 2	FM 3	FM 4	FM 5	FM 6
O						
DM 1	M	H	VH	M	M	MH
DM 2	M	MH	MH	M	ML	H
DM 3	M	H	VH	L	M	M
DM 4	MH	MH	VH	M	M	MH
DM 5	M	MH	VH	M	M	M
S						
DM 1	ML	H	MH	M	M	H
DM 2	ML	MH	MH	M	MH	H
DM 3	ML	H	MH	ML	MH	H
DM 4	M	H	MH	M	M	H
DM 5	M	H	MH	M	M	H
D						
DM 1	M	M	MH	VL	L	L
DM 2	ML	M	M	ML	ML	M
DM 3	ML	ML	MH	VL	L	L
DM 4	ML	M	MH	ML	L	L
DM 5	ML	M	M	VL	L	VL

**Table 8:** The evaluations to failure modes in the form of D numbers.

Failure Mode	O	S	D
FM 1	$\begin{array}{c} D(\{M\})=0.8,\\ D(\{MH\})=0.2 \end{array}$	$\begin{array}{c} D(\{ML\})=0.6,\\ D(\{M\})=0.4 \end{array}$	$\begin{array}{c} D(\{M\})=0.2,\\ D(\{ML\})=0.8 \end{array}$
FM 2	$\begin{array}{c} D(\{H\})=0.4,\\ D(\{MH\})=0.6 \end{array}$	$\begin{array}{c} D(\{H\})=0.8,\\ D(\{MH\})=0.2 \end{array}$	$\begin{array}{c} D(\{M\})=0.8,\\ D(\{ML\})=0.2 \end{array}$
FM 3	$\begin{array}{c} D(\{VH\})=0.8,\\ D(\{MH\})=0.2 \end{array}$	$\begin{array}{c} D(\{MH\})=1 \end{array}$	$\begin{array}{c} D(\{MH\})=0.6,\\ D(\{M\})=0.4 \end{array}$
FM 4	$\begin{array}{c} D(\{M\})=0.8,\\ D(\{L\})=0.2 \end{array}$	$\begin{array}{c} D(\{M\})=0.8,\\ D(\{ML\})=0.2 \end{array}$	$\begin{array}{c} D(\{VL\})=0.6,\\ D(\{ML\})=0.4 \end{array}$
FM 5	$\begin{array}{c} D(\{M\})=0.8,\\ D(\{ML\})=0.2 \end{array}$	$\begin{array}{c} D(\{M\})=0.6,\\ D(\{MH\})=0.4 \end{array}$	$\begin{array}{c} D(\{L\})=0.8,\\ D(\{ML\})=0.2 \end{array}$
FM 6	$\begin{array}{c} D(\{M\})=0.4,\\ D(\{MH\})=0.4,\\ D(\{H\})=0.2, \end{array}$	$\begin{array}{c} D(\{H\})=1 \end{array}$	$\begin{array}{c} D(\{L\})=0.6,\\ D(\{M\})=0.2,\\ D(\{VL\})=0.2 \end{array}$

**Table 9:** Risk ranking of failure modes by using the extended VIKOR method in [28].

	Failure Mode					
	FM 1	FM 2	FM 3	FM 4	FM 5	FM 6
By S	4	2	1	6	5	3
By R	5	3	2	6	4	1
By Q	5	3	1	6	4	2
